# Computational identification of receptor-like kinases “RLK” and receptor-like proteins “RLP” in legumes

**DOI:** 10.1186/s12864-020-06844-z

**Published:** 2020-07-03

**Authors:** Daniel Restrepo-Montoya, Robert Brueggeman, Phillip E. McClean, Juan M. Osorno

**Affiliations:** 1 Genomics and Bioinformatics Program, North Dakota State University, Fargo, ND 58105-6050 USA; 2 Department of Plant Sciences, North Dakota State University, Fargo, ND USA; 3 Department of Plant Pathology, North Dakota State University, PO Box 6050, Dept. 7660, Fargo, ND 58108 USA

**Keywords:** Dicots, Model plants, Resistance genes/proteins, Legumes, Plasma membrane receptors

## Abstract

**Background:**

In plants, the plasma membrane is enclosed by the cell wall and anchors RLK and RLP proteins, which play a fundamental role in perception of developmental and environmental cues and are crucial in plant development and immunity. These plasma membrane receptors belong to large gene/protein families that are not easily classified computationally. This detailed analysis of these plasma membrane proteins brings a new source of information to the legume genetic, physiology and breeding research communities.

**Results:**

A computational approach to identify and classify RLK and RLP proteins is presented. The strategy was evaluated using experimentally-validated RLK and RLP proteins and was determined to have a sensitivity of over 0.85, a specificity of 1.00, and a Matthews correlation coefficient of 0.91. The computational approach can be used to develop a detailed catalog of plasma membrane receptors (by type and domains) in several legume/crop species. The exclusive domains identified in legumes for RLKs are WaaY, APH Pkinase_C, LRR_2, and EGF, and for RLP are L-lectin LPRY and PAN_4. The RLK-nonRD and RLCK subclasses are also discovered by the methodology. In both classes, less than 20% of the total RLK predicted for each species belong to this class. Among the 10-species evaluated ~ 40% of the proteins in the kinome are RLKs. The exclusive legume domain combinations identified are B-Lectin/PR5K domains in *G. max*, *M. truncatula*, *V. angularis*, and *V. unguiculata* and a three-domain combination B-lectin/S-locus/WAK in *C. cajan*, *M. truncatula*, *P. vulgaris*, *V. angularis*. and *V. unguiculata*.

**Conclusions:**

The analysis suggests that about 2% of the proteins of each genome belong to the RLK family and less than 1% belong to RLP family. Domain diversity combinations are greater for RLKs compared with the RLP proteins and LRR domains, and the dual domain combination LRR/Malectin were the most frequent domain for both groups of plasma membrane receptors among legume and non-legume species. Legumes exclusively show Pkinase extracellular domains, and atypical domain combinations in RLK and RLP compared with the non-legumes evaluated. The computational logic approach is statistically well supported and can be used with the proteomes of other plant species.

## Background

Plants have evolved a surveillance system that is continuously monitoring a broad range of stimuli, including tissue damage or altered developmental processes, or establishing a symbiotic interaction. They commonly use pattern recognition receptors (PRR) to perceive 1) microbe-, pathogen-, or damage-associated molecular patterns (MAMP/PAMP/DAMP); 2) virulence factors; 3) secreted proteins; and 4) processed peptides directly or indirectly with specific molecular signatures [1]. These membrane-bound PRR are receptor-like kinases (RLK) or receptor-like proteins (RLP). The two receptor classes are located on the plant plasma membrane and are known as modular transmembrane proteins [2]. In contrast, the intracellular resistance proteins such as the nucleotide binding site-leucine-rich repeat proteins (NB-LRR or NBS-LRR) are encoded by the so-called resistance genes (R genes) and have been targeted to elicit a resistance response to pathogens [3]. These intracellular resistance genes are out of the scope of this study.

R genes are broadly categorized into eight classes based on their motif organization and membrane domains [4]. Following this classification system and depending on their protein structure, three belong to the RLK and RLP categories, such as the gene resistance to *Cladosporum fulvum*: Cf-9, Cf-4, and Cf-2 (class III); the gene resistance to *Xanthomonas oryzae* – race 6: Xa21 (resistance to) (class IV); and *Verticillium* wilt resistance genes: Ve1 and Ve2 (class V) [4]. Proteins such as the polygalacturonase-inhibiting protein (PGIP) also play an important role for certain defense proteins even though they are not directly involved in pathogen recognition or activation of any defense genes [4]. In contrast, the PRRs confer a broad-spectrum resistance and are modular transmembrane RLK or RLP proteins, and their recognition is based on a set of conserved molecules [5]. Most characterized RLK/RLP are involved in defense/resistance processes in plants (Additional file 1: Table S1) or are actively involved in cell growth and development, such as floral organ abscission (*A. thaliana* – HAESA) [6], meristem development (*A. thaliana* – CLAVATA) [7], self-incompatibility (MPLK) [8], abscission (CST) [9], stomatal patterning (TMM) [10], and embryonic patterning (SSP) [11].

RLK and RLP are structurally identified by the presence of motifs involved in the protein transport system, such as signal peptide. The transmembrane helices anchors the RLK/RLP to plasma membrane [12]. The extracellular domains, or ectodomains, are functional regions located outside of the cell and initiate contact with other molecules or surfaces and lead to signal transduction [2, 3, 5, 13–17]. Among the ectodomains, the LRR are a component of N-glycosylated plant proteins, and many N-glycosylation acceptor sequences are present in all ectodomains [18]. The C (Carbohydrate-binding protein domain)/G (S-receptor-like or S-locus)/L (L-like lectin domain), LysM (Lysin Motif), and malectin classes of lectins are key players in plant immunity [19]. The C/G/L lectins are omnipresent in plants [20]. LysM receptors are the most studied lectins, and 15 RLK-LysM and five RLP-LysM have been functionally characterized [21]. These proteins are known to play an essential role in plant defense signaling and inducing symbiosis. Among these proteins are NFR1 (Nod factor receptor 1) [22], NFR5 (Nod factor receptor 5) [22], LYK3 (putative Medicago ortholog of NFR1) [23], and NFP (LysM protein controlling Nod factor perception) [24], that recognize lipochitooligosaccharide nod factors [25]. Malectin-like domain-containing and FERONIA protein (FER or protein Sirene) receptors are recognized as critical regulators of cell growth and appear to function as surveyors of cell-wall status [26].

Other ectodomain families include the PR-5 family (Pathogenesis-related protein 5), composed of thaumatin-like proteins (TLPs) are responsive to biotic and abiotic stress and are widely studied in plants [27]. Cell-wall-associated kinases (the “WAK” family) and their roles in signal transduction and pathogen stress responses arose from studies of the model plant species *A. thaliana* [28, 29]. The hallmark of a WAK is the presence of epidermal growth factor-like repeats (“EGF”) in the extracellular domain [2, 3]. In contrast to the WAK, the evolution of the tumor necrosis factor/tumor necrosis factor receptor superfamily (“TNF/TNFR”) is complicated and not well understood [30], and even though the TNFR domain is conserved in dicots and monocots, this domain family has distinctive characteristics among taxonomic families [31]. The stress-antifung domain family (known as DUF26 – Domain of Unknown Function) belongs to the cysteine-rich receptor-like protein kinases that form one of the largest groups of RLK in plants [32]. The structural details of RLK and RLP are reviewed by different authors [3, 13, 14, 33, 34].

RLKs and RLPs typically display high target specificity and selectivity [3, 35]. This provided an opportunity to understand how plants differentiate and distinguish favorable and harmful stimuli, as well as how various receptors coordinate their roles under variable environmental conditions [3]. The RLK family belongs to the protein kinase superfamily that has expanded in the flowering plant lineage, in part through recent duplications. Particularly, the flowering plant protein kinase repertoire known as “kinome,” (a term coined by Manning et al., 2002 [36]), describes the catalog of protein kinases in a genome and is significantly larger (600 to 2500 members) than the kinome in other eukaryotes. This large variation among organisms is principally due to the expansion and contraction of a few families; more than 60% of the kinome belongs to the receptor-like kinase/Pelle flowering plants family [37, 38]. The kinase domains can be divided into RD and non-RD families based on the presence or absence of an arginine (R) located before a catalytic aspartate (D) residue [39]. Non-RD kinases lack the strong autophosphorylation activities of RD kinases and display lower enzymatic activities [40]. Non-RD kinases are associated with innate immune receptors that recognize conserved microbial signatures [39]. Computational and comprehensive tools related to the prediction and analysis of resistance genes, such as RLKs or RLPs, could potentially support plant breeders/geneticists to identify candidate resistance genes to facilitate the understanding of new resistance sources and mechanisms, which may be useful for crop improvement [41].

The RLPs function with RLKs to regulate development and defense responses. The similarities between the structure of RLPs and RLKs and their functional relationships suggest that RLKs with novel domain configurations may have evolved through fusions of an RLP and RLK [35, 42]. Since most RLP are membrane-spanning proteins, they most likely are integral components of extracellular signaling networks. Fusions between ancestral RLP and RLK/Pelle kinases could, therefore, have led to novel signal transduction pathways by linking ligand perception to different downstream kinase mediated signaling pathways. Alternatively, fusions may simply have occurred between RLP and RLK/Pelle that were already components of the same signaling networks [35].

In recent years, more than 20 studies to computationally identify cytoplasmic resistant proteins (mostly NBS-LRR) from different plant species have been published [43, 44]. Due to the diversity of extracellular receptor domains, which makes them harder to characterize compared to cytoplasmic resistant proteins, efforts to identify and characterize RLKs/RLPs computationally have been limited (see review by Sekhwal and colleagues [43]). These genomic studies targeted many plant species [45], including *Arabidopsis* [46], *Arabidopsis* and rice (*Oryza sativa* L.) [47], grape (*Vitis vinifera* L.) [48], and tomato (*Solanum lycopersicum* (L.) H. Karst) [49], among others. To date, the strategies used similar computational approaches, but no standardized computational tools or annotation criteria were followed. Thus, the results from different studies are not necessarily comparable [43]. Furthermore, the establishment of robust, independent, and highly diverse data with multiple examples is required to evaluate the performance of the strategies and tools published [50, 51].

Recently, legume genomics tools have expanded because of advancements in high-throughput sequencing and genotyping technologies resulting in reference genome sequences for many legume crops. This allowed the identification of structural variations and enhanced the efficiency and resolution of large-scale genetic mapping and marker-trait association studies for legumes [52, 53]. Legumes are considered the second most important family of crop plants after the grass family based on their economic relevance. Approximately 27% of world crop production is composed of grain legumes, providing 33% of human dietary protein, while pasture and forage legumes are fundamental for animal feed [54]. To date, no RLK and RLP comparative genomic analyses have been published that explores the genomes of soybean (*Glycine max* (L.) Merrill; GM [55], common bean (*Phaseolus vulgaris* L.; PV) [56], barrel medic (*Medicago truncatula* L.; MT) [57], mungbean (*Vigna radiata* (L.) R. Wilczek; VR) [58], cowpea (*Vigna unguiculata* L. Walp; VU) [59], Adzuki bean (*Vigna angularis* var. *Angularis*; VA) [60], and pigeonpea (*Cajanus cajan* L.; CC) [61].

This study describes the computational identification of receptor-like proteins and receptor-like kinase proteins and probable resistance RLK-nonRD proteins in legumes using probabilistic methods [62–64]. The computational identification of these plasma membrane receptors is based on the prediction of presence/absence of a signal peptide, transmembrane helix motif/s, and extracellular and intracellular domains. The domain combination was considered as the presence of two or more domains that may occur in a protein and were evaluated to illustrate the domain mixture. The performance of the proposed strategy was evaluated with experimentally-validated RLK (*n* = 63) and RLP (*n* = 27) proteins (Additional file 1: Table S1), and the RLK/RLP identification was applied on protein datasets that belong to the seven legume genomes mentioned above. Also, three non-legume model plant species were included to enrich the analysis due to the high quality of its genomic annotation. These species are *Arabidopsis thaliana* (L. Heynh; AT) [65]; tomato (*S. lycopersicum*; SL) [49]; and common grape (*V. vinifera*; VV) [66], which represents the basal rosid lineage and has ancestral karyotypes that facilitate comparisons across major eurosids [66, 67].

## Results

### Performance prediction of RLK and RLP

The independent performance evaluation of the computational strategy identified 56 out of a total 63 RLK proteins as true RLK, and the remaining proteins were not detected and considered as false negatives. In contrast, 23 out of the total 27 RLP proteins were classified as true RLP, and the remaining proteins were not detected and classified as false negatives. Lastly, none of the 96 proteins belonging to the cytoplasmic R gene classes were classified as RLKs or RLPs (Additional file 2: Table S2). Based on these results, the performance predictive measures were calculated (Table 1).

**Table 1:** Performance evaluation

**Measure**	**RLK**	**RLP**
Sensitivity	0.88	0.85
Specificity	1	1
Matthews correlation coefficient	0.91	0.91

Non-redundant datasets used for the performance evaluation are RLK, n:63; RLP, n:27; and Other R genes, *n* = 96. The Additional file 1: Table S1 - lists the experimentally-validated proteins used for this evaluation including information about its prediction condition (RLK, RLP, and cytoplasmic resistance proteins), and the Additional file 2: Table S2 – provides a performance evaluation summary

**Fig. 1:**
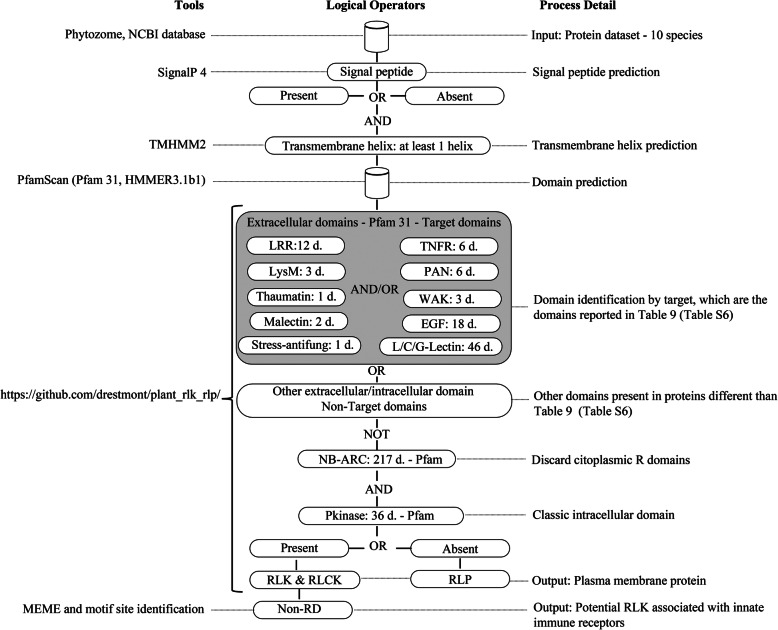
Computational strategy followed to identify RLK and RLP

This evaluation established a minimum set of conditions to classify the RLK or RLP protein classes. RLK- and RLP-predicted proteins must have at least one transmembrane helix with the presence of at least one extracellular domain (LRR, L/C/G-Lectin, LysM, PR5K, thaumatin, WAK, malectin, EGF, or stress-Antifung). Additionally, for RLK, the presence of an intracellular Pkinase domains is also required, and for RLP, the absence of Pkinase and NB-ARC domains is required; these logic conditions are stated in Fig. 1.

### Summary of predicted RLK and RLP

Based on the number of RLKs and RLPs identified among all species, about 3% or less of the total proteins per species belong to these classes of membrane bound receptor-like proteins. Specifically, for legumes, the percentage ranged from 0.9 to 2.3% for RLKs and 1.4 to 1.7% for non-legumes. The RLP percentage ranges from 0.3 to 0.7% for legumes, and 0.5 to 0.6% for non-legumes species. The species analysis evaluated 447,948 proteins, with 351,491 from legumes, and 96,457 from non-legumes. Almost 9.4% of the legume and 9.7% of the non-legume predicted proteins had a predicted signal peptide, and 4.3% of legumes and 4.4% of non-legumes had at least one transmembrane helix above the threshold. For the subset of proteins without a predicted signal peptide, 16.6% of legumes and 17.9% of non-legumes reached the TMHMM cut-off. Among the total number of proteins evaluated, 1.9% of legumes and 1.5 of non-legumes belong to the RLK class of proteins, and 0.5% of legumes and 0.5% of non-legumes belong to the RLP class (Table 2). Also, the number of RLK proteins identified as non-RD, which are potentially kinases associated with innate immune receptors, are reported in Table 2 footnote (Additional file 3: Table S3), and the differentiated proteins identified by species for RLK are in the Additional file 4: Table S4 and for RLP are in the Additional file 5: Table S5.

**Table 2:** Summary of total number of RLK and RLP identified across legumes/non-legumes

		Signal peptide			Transmembrane helices		RLK/RLP proteins identified per species			
Species	Total proteins reported	Pre/Abs	Number of proteins	%	Number of proteins	%	RLK^a^	% RLK	RLP^a^	% RLP
*C. cajan*	48,331	P	2679	5.5	1031	2.1	197		62	
		A	45,652	94.4	5760	11.9	253		80	
						total	450	0.9	142	0.3
*G. max*	88,647	P	8125	9.1	3934	4.4	1182		282	
		A	80,522	90.8	15,459	17.4	682		186	
						total	1864	2.1	468	0.5
*M. truncatula*	62,319	P	6251	10.0	2961	4.7	647		196	
		A	56,068	89.9	10,383	16.6	413		167	
						total	1060	1.7	363	0.6
*P. vulgaris*	36,995	P	4120	11.1	1895	5.1	571		138	
		A	32,875	88.8	6349	17.1	271		79	
						total	842	2.3	217	0.6
*V. angularis*	37,769	P	3570	9.4	1681	4.4	557		124	
		A	34,199	90.5	6364	16.8	278		91	
						total	835	2.2	215	0.6
*V. radiata*	35,143	P	3450	9.8	1584	4.5	505		142	
		A	31,693	90.1	5934	16.8	265		99	
						total	770	2.2	241	0.7
*V. unguiculata*	42,287	P	4698	11.1	2105	4.9	660		190	
		A	37,589	88.9	7962	18.8	332		104	
						total	992	2.3	294	0.7
*V. vinifera*	26,346	P	2043	7.7	842	3.2	269		99	
		A	24,303	92.2	4980	18.9	174		73	
						total	443	1.7	172	0.6
*A. thaliana*	35,386	P	4088	11.5	1935	5.4	408		121	
		A	31,298	88.4	5784	16.3	147		51	
						total	555	1.6	172	0.5
*S. lycopersicum*	34,725	P	3258	9.3	1480	4.2	316		107	
		A	1467	90.6	5727	16.4	160		54	
						total	476	1.4	161	0.5

For each species, the results were distinguished by the present “P” and absent “A” of signal peptide and follow the logic flow presented in Fig. 1. ^a^Non-redundant data reported. For the RLK-nonRD, the results per species are: *A. thaliana*: 48 proteins (8.6%), *C. cajan*: 61 proteins (13.6%), *G. max*: 223 proteins (11.9%), *M. truncatula*: 194 proteins (18.3%), *P. vulgaris*: 124 proteins (14.7%), *S. lycopersicum*: 83 proteins (17.4%), *V. angularis*: 122 proteins (14.6%), *V. radiata*: 113 proteins (14.7%), *V. unguiculata*: 158 proteins (15.9%), and *V. vinifera*: 59 proteins (13.3%). RLK-nonRD IDs are reported in the Additional file 3: Table S3. The kinome (total set of proteins with a kinase in a genome) per species was calculated and the results for the species are CC: 1268 p. (35.5% - RLK), GM: 4497 p. (41.4% - RLK), MT: 2281 p. (46.6% - RLK), PV: 1888 p. (44.7% - RLK), VA: 1898 p. (44% - RLK), VR: 1772 p. (43.5% - RLK), VU: 2090 p. (47.5% - RLK), VV: 1064 p. (41.7% - RLK), AT: 1431 p. (38.9% - RLK), and SL: 1194 p. (39.9% - RLK)

Based on the Pfam clans and families of domains of known function used to filter the identified RLKs and RLPs, the computational strategy allowed for the identification of extra domains present in the predicted proteins (Additional file 6: Table S6). For the RLK proteins reported in Table 3, the approach identified, besides a Pkinase domain, up to four combinations of functional domains (located extra or intracellularly). Almost all the classical domains reported by different authors [3, 13, 14, 33, 34] for RLKs and RLPs were identified, the exception was the TNFR domain in which the in-house scripts (https://github.com/drestmont/plant_rlk_rlp/) did not identify its present in any of the datasets; however, when reviewing the approach, it was found that the TNFR domains predicted by Pfam 31, HMMER, and PfamScan did not reach the minimum cut-offs in the prediction process followed. All species evaluated had proteins with at least one extra domain (Additional file 7: Table S7).

**Table 3:** Receptor-like kinases identified by extracellular domains across the species

Domain class	Domain combinations	Species									
		CC	GM	MT	PV	VA	VR	VU	VV	AT	SL
LRR	lrr	134	579	324	239	254	249	301	136	180	198
G-lectin: combination of ectodomains	s-locus	2	1	1	1	1	1	3	2	0	0
	b-lectin	7	20	25	12	12	14	15	7	2	7
	b-lectin/pan	2	9	7	12	2	5	17	2	1	5
	s-locus/pan	5	10	4	5	1	0	7	18	2	0
	b-lectin/s-locus	11	24	14	15	14	18	15	7	2	10
	b-lectin/s-locus/pan	31	146	131	41	53	44	96	12	33	42
L-Lectin	l-lectin	24	66	46	38	35	36	42	20	44	22
C-lectin	c-lectin	1	4	1	1	1	1	1	1	1	1
Lectin	lysM	7	27	16	14	11	12	13	5	5	8
Lectin (Feronia)	malectin	29	99	54	82	58	50	60	29	36	22
Thaumatin (Osmotin)	pr5k	0	0	0	0	0	0	0	0	2	0
WAK	wak	11	66	33	41	45	39	46	14	27	17
	egf	1	4	0	1	0	0	1	2	0	2
	wak/egf	5	10	16	6	3	7	8	7	4	7
DUF26 recently renamed	stress_antifung	28	173	66	70	57	58	90	22	45	15
Classically related to G-lectin	pan	5	10	1	2	2	0	1	10	0	0
Combination of different domain ectodomains identified	lrr/malectin	12	63	66	32	30	19	28	26	47	7
	pan/wak	0	0	0	0	0	0	0	1	0	0
	s-locus/wak	0	0	0	0	0	2	0	1	0	0
	b-lectin/pr5k	0	1	3	0	1	0	1	0	0	0
	b-lectin/s-locus/wak	2	0	1	2	3	0	3	0	0	0
	b-lectin/s-locus/pan/wak	0	8	2	2	2	2	5	1	0	1
	pan/s-locus/wak	0	0	0	0	0	0	0	1	0	0
RLK - pkinase	rlk – non-target ectodomain	4	11	6	5	3	5	6	18	8	5
Combination of ectodomains Identified RLCK with/without ectodomains	rlk - not ectodomains	30	180	74	87	93	72	86	80	25	28
	rlck extra domain	8	7	6	6	10	3	5	5	5	9
	rlck only pkinase	91	346	163	128	144	133	142	16	86	70

For each species, the results were merge by present “P” and absent “A” of signal peptide. All possible domain combinations were explored and are reported in the “Domain combinations” column (proteins reported are non-redundant). *A. thaliana*: AT, *C. cajan*: CC, *G. max*: GM, *M. truncatula*: MT, *P. vulgaris*: PV, *S. lycopersicum*: SL, *V. angularis*: VA, *V. radiata*: VR, *V. unguiculata*: VU, and *V. vinifera*: VV (Table A4). RLCK: Only kinase domain identified. All proteins reported in this table have at least one transmembrane helix. Extra: proteins that have the presence/absence of signal peptide, at least one transmembrane helix, a Pkinase and other extracellular/intracellular domains different than LRR, L/C/G-Lectin, LysM, Pr5k-Thaumatin, WAK, Malectin, EGF or Stress-Antifung were only considered for the combination identification analysis, but other domains reported in Table A7 named as “non-target” domains could be present

The G-lectin class of proteins reported in Table 3 is typically composed of three domains (B-lectin/S-locus/PAN); however, different combinations of these three domains were identified. C-lectin is a rare domain, and only soybean species showed more than one C-lectin protein. The WAK is typically composed of two domain classes (WAK/EFG), and such proteins possessed one or the other domain. The dual domain combination LRR/Malectin is the most frequent among the atypical dual combinations. Also, atypical domain combinations with a low frequency among the species were identified. Among the legumes, these were the B-Lectin/PR5K combination in GM, MT, VA, and VU and a three-domain combination of B-lectin/S-locus/WAK only in CC, MT, PV, VA, and VU. Among non-legumes, the uncommon dual combinations PAN/WAK and PAN/S-locus/WAK were only found in VV. The only uncommon domain combination found in both legumes/non-legumes was S-locus/WAK in VV and VR.

A four-domain combination, consisting of B-lectin/S-locus/PAN/WAK domains, was present GM, MT, PV, SL, VA, VR, VU, and VV species. Across all legume/non-legume species, the LRR ectodomain class was the most frequent domain per species. The computational classification strategy also discovered RLK proteins with no other domains and some proteins with the additional domains beyond the signal peptide, transmembrane helix, and Pkinase domains. In the case of the RLCK, the proteins that belong to this class are the kinases without signal peptide, but with a transmembrane helix. The RLCKs without another plasma membrane attachment domain were not predicted (Table 3).

For the RLP extracellular domain identification and domain combinations reported in Table 4, the computational approach allowed the identification of up to three possible combinations of additional functional domains (which could be located extra or intracellularly) in the proteins evaluated; however, all combinations correspond to the typical combinations reported in Additional file 7: Table S7, such as the G-lectin (B-lectin/S-lectin/PAN) present in legumes/non-legumes, the classic WAK/EGF only present in CC and VV (legume/non-legume), and the LRR/Malectin present in all species evaluated. However, the three cases mentioned were of a low frequency compared with other domains, such as LRR or Stress-antifung. As in RLK, for RLP, the most abundant ectodomain for all species was the LRR, and no RLP proteins were contained a C-lectin or TNFR domain.

**Table 4:** Receptor-like proteins identified by extracellular domains across the species

Domain details	Domain combinations	Species									
		CC	GM	MT	PV	VA	VR	VU	VV	AT	SL
LRR	lrr	69	247	225	107	104	138	171	78	71	67
G-lectin: combination of ectodomains identified	s-locus	0	0	0	0	0	0	0	1	0	0
	b-lectin	1	5	2	5	3	2	5	8	4	1
	s-locus/pan	0	0	0	0	0	0	0	2	0	0
	b-lectin/s-locus	0	1	1	0	0	0	0	2	0	1
	b-lectin/s-locus/pan	2	3	3	3	1	2	5	4	2	1
L-lectin	l-lectin	31	88	54	35	39	33	34	27	34	36
Lectin	lysM	3	7	8	5	3	5	4	3	2	4
Lectin (Feronia)	malectin	5	12	7	3	5	3	5	8	7	3
Thaumatin (Osmotin)	pr5k	7	28	19	13	16	17	20	7	15	16
WAK	wak	5	14	16	8	9	12	11	10	8	12
	egf	4	17	5	6	8	5	8	3	5	3
	wak/egf	1	0	0	0	0	0	0	2	0	0
DUF26 recently renamed	stress_antifung	12	34	15	22	22	19	24	9	23	14
Classically related to G-lectin	pan	1	3	3	1	1	1	3	3	0	0
Combination of target ectodomains	lrr/malectin	1	9	5	9	4	4	4	5	1	3

For each species, the results were distinguished by present “P” and absent “A” of signal peptide, all possible domain combinations were explored and are reported in the “Domain combinations” column. Proteins reported are non-redundant. *A. thaliana*: AT, *C. cajan*: CC, *G. max*: GM, *M. truncatula*: MT, *P. vulgaris*: PV, *S. lycopersicum*: SL, *V. angularis*: VA, *V. radiata*: VR, *V. unguiculata*: VU, and *V. vinifera*: VI. All proteins reported in this table have at least one transmembrane helix. Other domains reported in Table A8 named as “non-target” domains could be present

### Summary of the presence and prevalence of functional domains

**Fig. 2:**
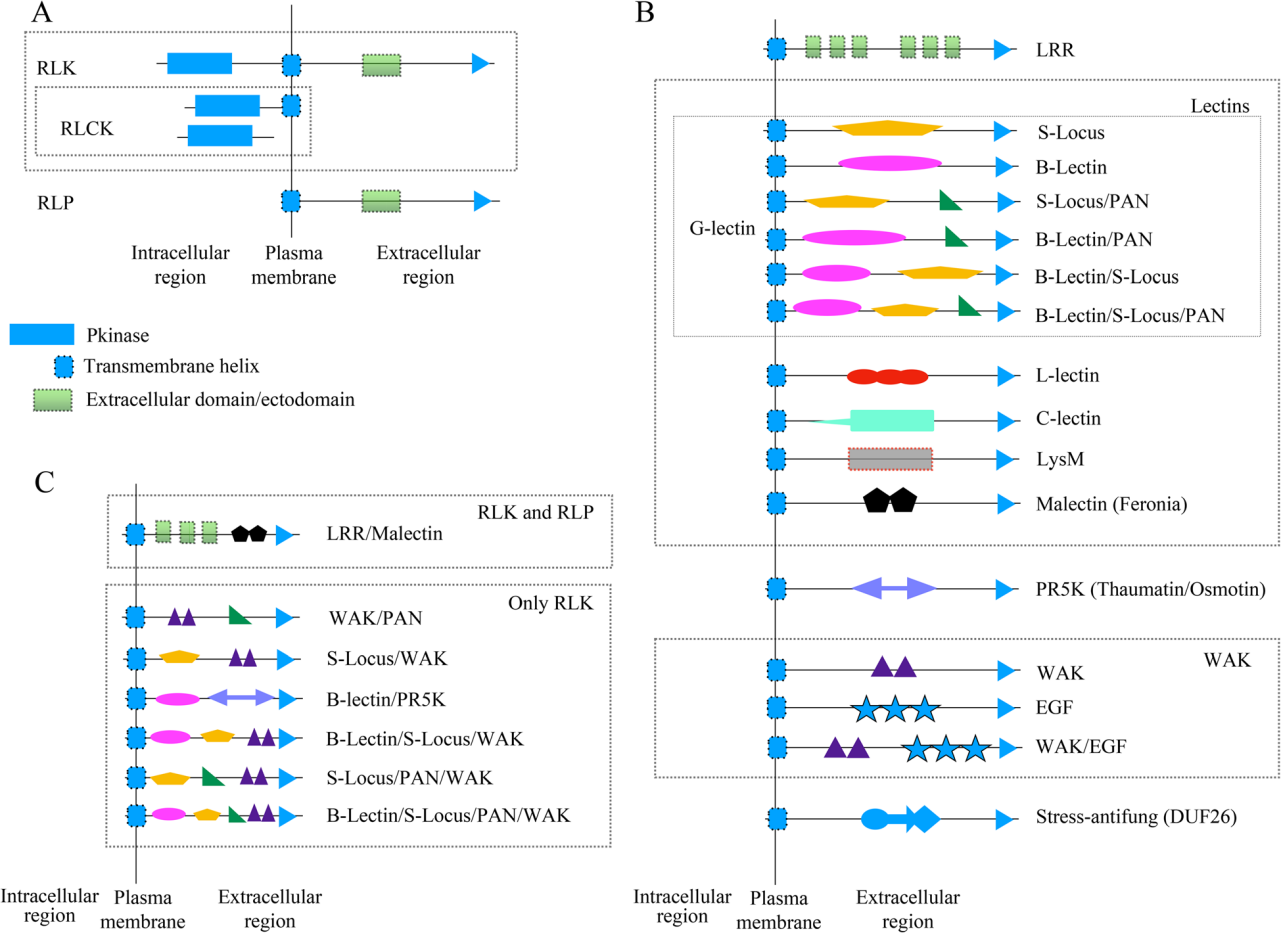
Summary of the extracellular domains identified in RLK/RLP. The domains in this figure resume the domains and the combinations identified. A. Classical RLK/RLP protein structure. B. Ectodomains identified that are also reported by the scientific community (Tables 1 and 2). C. Ectodomain combinations identified in RLK/RLP. In B and C, the ectodomains are only represented, in the RLK cases all proteins must have an intracellular Pkinase

As a result of the identification process for RLK and RLP are summarized in Fig. 2, the specific domains that belong to the clans and families (Additional file 6: Table S6, Additional file 7: Table S7, and Additional file 8: Table S8) are reported in Tables 5, and 6. Table 7 shows the domains identified in the RLK and RLP proteins (Additional file 1: Table S1) used to evaluate the performance of the plasma membrane identification process.

**Table 5:** Summary of domains present on the RLK proteins predicted

Clan or domain	Pfam domain name ID	Species									
		CC	GM	MT	PV	VA	VR	VU	VV	AT	SL
Pkinase	Ins_P5_2-kin						x			x	
	RIO1			x						x	
	Pkinase	x	x	x	x	x	x	x	x	x	x
	PI3_PI4_kinase	x							x	x	
	Pkinase_Tyr	x	x	x	x	x	x	x	x	x	x
	Choline_kinase		x	x					x	x	x
	ABC1	x	x	x	x	x	x	x	x	x	x
	Pkinase_C	x	x								
	PIP5K		x								x
	WaaY			x							
	APH				x	x	x	x			
LRR	LRRNT_2	x	x	x	x	x	x	x	x	x	x
	LRR_8	x	x	x	x	x	x	x	x	x	x
	LRR_1	x	x	x	x	x	x	x	x	x	x
	LRR_4	x	x	x	x	x	x	x	x	x	x
	LRR_6	x	x	x	x	x	x	x	x	x	x
	LRR_2		x			x		x			
	LRR_5				x	x	x	x			x
L-Lectin	Lectin_legB	x	x	x	x	x	x	x	x	x	x
C-Lectin	Lectin_C	x	x	x	x	x	x	x	x	x	x
G-Lectin	B_lectin	x	x	x	x	x	x	x	x	x	x
	S_locus_glycop	x	x	x	x	x	x	x	x	x	x
PAN	PAN_2	x	x	x	x	x	x	x	x	x	x
	PAN_1		x					x			x
LysM	LysM	x	x	x	x	x	x	x	x	x	x
PR5K	Thaumatin		x	x		x		x		x	
WAK	WAK_assoc	x	x	x	x	x	x	x	x	x	x
	WAK	x	x	x					x	x	x
	GUB_WAK_bind	x	x	x	x	x	x	x	x	x	x
Malectin	Malectin_like	x	x	x	x	x	x	x	x	x	x
	Malectin	x	x	x	x	x	x	x	x	x	x
EGF	EGF_CA	x	x	x	x	x	x	x	x	x	x
	EGF		x	x							
	EGF_3			x	x	x	x	x			x
Stress-antifung (DUF26)	Stress-antifung	x	x	x	x	x	x	x	x	x	x

Present: X

**Table 6:** Summary of domains present on the RLP proteins predicted

Clan or Domain	Domain name	Species									
		CC	GM	MT	PV	VA	VR	VU	VV	AT	SL
LRR	LRR_8	x	x	x	x	x	x	x	x	x	x
	LRR_1	x	x	x	x	x	x	x	x	x	x
	LRRNT_2	x	x	x	x	x	x	x	x	x	x
	LRR_2			x						x	
	LRR_4	x	x	x	x	x	x	x	x	x	x
	LRR_6	x	x	x	x	x	x	x	x	x	x
	LRR_9	x									
	LRR_5								x		
L-Lectin	Gal-bind_lectin	x	x	x	x	x	x	x	x	x	x
	Glyco_hydro_32C	x	x	x	x	x	x	x		x	x
	XET_C	x	x	x	x	x	x	x	x	x	x
	Lectin_legB	x	x	x	x	x	x	x	x	x	x
	Glyco_hydro_16	x	x	x	x	x	x	x	x	x	x
	Calreticulin	x	x	x	x	x	x	x	x	x	x
	SPRY	x	x	x	x	x	x	x			
	Alginate_lyase2		x						x		
G-Lectin	B_lectin	x	x	x	x	x	x	x	x	x	x
	S_locus_glycop	x	x	x	x	x	x	x	x	x	x
PAN	PAN_2	x	x	x	x		x	x	x	x	x
	PAN_1	x	x	x	x	x	x			x	
	PAN_4	x	x	x	x	x	x	x			
LysM	LysM	x	x	x	x	x	x	x	x	x	x
Thaumatin (PR5K)	Thaumatin	x	x	x	x	x	x	x	x	x	x
WAK	WAK_assoc	x	x	x	x	x	x	x	x	x	x
	WAK								x	x	
	GUB_WAK_bind	x	x	x	x	x	x	x	x	x	x
Malectin	Malectin_like	x	x	x	x	x	x	x	x	x	x
	Malectin		x	x	x				x		x
EGF	EGF_alliinase	x	x					x	x	x	x
	cEGF	x	x	x	x	x	x	x	x	x	x
	EGF_CA	x		x					x		
	EGF_2		x	x	x	x	x	x			x
Stress-antifung	Stress-antifung	x	x	x	x	x	x	x	x	x	x

X: Present

**Table 7:** Summary of domains identified in the validation dataset

Clan or Domain	Domain or Family	RLK	RLP
PKinase	Pkinase_Tyr	X^a^	
	Pkinase	X	
LRR	LRR_8	X	X
	LRRNT_2	X	X
	LRR_1	X	X
	LRR_4	X	X
	LRR_6	X	X
L-Lectin	Lectin_legB	X	
G-Lectin	B_lectin	X	
	PAN_2	X	
	S_locus_glycop	X	
LysM	LysM	X	X
PR5K	Thaumatin	X	
WAK	GUB_WAK_bind	X	
	WAK	X	
Malectin	Malectin_like	X	
EGF	EGF_CA	X	
Stress-Antifung	Stress-antifung	X	
DUF3403	DUF3403	X	
CL0384	GDPD	X	

^a^X: Present. Source: Table A1: the list of experimentally-validated proteins used for this evaluation were RLK, n:63 and RLP, n:27

The domains in this figure resume the domains and the combinations identified. A. Classical RLK/RLP protein structure. B. Ectodomains identified that are also reported by the scientific community (Additional file 7: Table S7 and Additional file 8: Table S8). C. Ectodomain combinations identified in RLK/RLP. In B and C, the ectodomains are only represented, in the RLK cases all proteins must have an intracellular Pkinase.

Table 5 shows the domains identified in the predicted RLK, and Table 6 shows the domains identified in the predicted RLP. In the target domains (domains classically reported as present in RLK and RLP proteins) identified on the experimentally-validated RLK and RLP proteins (Additional file 1: Table S1), almost all of the domains were identified for the RLKs with the exception of the C-Lectin and TNFR domains. Also, two additional domains (DUF3403 and CL0384) were found in the sequences of the proteins evaluated. For the evaluated RLPs, only domains belonging to LRR and LysM were identified. Regarding the ectodomain classes reported for RLKs and RLPs (Table A1), the expected domains were identified using the strategy implemented in this study (Table 7).

Among the predicted RLKs, 125 Pfam domains (Table 5 and Additional file 9: Table S9) were classified, with 35 domains (Table 5) belonging to the “target domains” (Additional file 6: Table S6 and Additional file 7: Table S7). The remaining domains are included in Additional file 9: Table S9. Independent of the Pkinase domains, which are cytoplasmically located, the other domains could be present either extra- or intracellularly. Comparing the domains identified in the predicted RLKs and RLPs against the target Pfam domains (Additional file 6: Table S6) for the identification of extra/intracellular domains, 10 out of 35 Pkinase domains, 7 out of 12 LRR domains, 1 out of 43 L-Lectin domains, 1 out of 1 C-Lectin domains, 5 out of 8 G-Lectin domains, 1 out of 3 LysM domains, 1 out of 1 PR5K domain, 3 out of 3 WAK domains, 2 out of 2 Malectin domains, 3 out of 18 EGF domains, and 1 out of 1 Stress-antifung domain were identified. Also, with the exception of the TNFR, all families and domains reported in Table 1 were identified in all 10 species. Of the non-target domains, which are considered additional domains that are different to the classically reported in RLK and RLP proteins, a total of 90 were identified (Additional file 9: Table S9), the most prevalent were RCC1_2, DUF3403, Ribonuc_2-5A, NAF, DUF3660, and Glyco_hydro_18, all of which were present in at least eight species (legumes/non-legumes); the remaining domains (84 in total) were present in two or fewer species.

For the entire set of domains identified in the RLPs, 71 domains (Table 6 and Additional file 10: Table S10) were identified, 33 (Table 6) belong to the “target domains” (Additional file 6: Table S6 and Additional file 7: Table S7), and the remaining domains are reported in Additional file 10: Table S10. All domains present in this dataset are extracellularly located. Comparing the domains identified with the total of Pfam (31 version) clans and families evaluated (Additional file 6: Table S6) used to identify extra/intracellular domains (Fig. 1), the RLK and RLP predicted for the 10-species evaluated allowed to identified 8 out of 12 LRR domains, 8 out of 43 L-Lectin domains, 5 out of 8 G-Lectin domains, 1 out of 3 LysM domains, 1 out of 1 PR5K domain, 3 out of 3 WAK domains, 2 out of 2 Malectin domains, 4 out of 18 EGF domains, and 1 out of 1 Stress-antifung domain were identified. Also, with the exception of C-Lectin and the TNFR family, all families and domains are reported in Additional file 7: Table S7. Of the non-target domains (38 in total Additional file 10: Table S10), the most prevalent were DUF2854, Glyco_hydro_32N, DUF3357, Alliinase_C, Galactosyl_T, zf-RING_2, PA, Peptidase_M8, and Exostosin, all of which were present in at least six species; the remaining domains (29 in total) were present in three or fewer legumes/non-legumes species.

## Discussion

The performance evaluation of the computational approach to predict RLK and RLP proteins were previously shown to be associated with biotic resistance. The quality of the validation dataset (Additional file 1: Table S1) is ideal because the data come from diverse species and are independent, experimentally-validated, and non-redundant. Based on the legume/non-legume results, the RLK proteins are more diverse in terms of domains compared to RLP proteins (Table 7). With respect to sensitivity and specificity, the sensitivity measure of the process suggests it was able to classify a protein as RLK/RLP with only a few false negatives. The specificity measure evaluated the ability of the approach to correctly classify a protein as non-RLK/RLP. The combined results indicate a greater ability to identify few false positive proteins. Based on the Matthews correlation coefficient, the performance evaluation reports a very strong positive value (0.91), which suggests the approach is ideal for RLK/RLP identification [50].

As for the RLK/RLP prediction requirements described in Fig. 1, the prediction and identification of RLK using the logic sum of conditions was a restively simple work flow. The Pkinase domain is required RLK proteins, in contrast with the logic sum of conditions that a protein needs to be classified as an RLP. Interestingly, for the last plasma membrane class mentioned, apart from the conditions that proteins must meet to belong to the RLP class, one factor that improves the confidence of the prediction and reduces false positive protein is the exclusion of cytoplasmic resistance genes which could be confounded with RLP. This is accomplished by excluding proteins with a NB-ARC domain.

Of the total plasma membrane proteins reported in Table 2, the results for *G. max* had the largest set of RLKs and RLPs compared with all other species, a result most probably due to its recent whole genome duplication about 13 MYA [68, 69]. Such duplications are the main mechanism for the expansion of the protein kinase superfamily in plants [37]. Regarding the RLK-nonRD class, with the exception of the non-legume AT (8.6%), the other legume/non-legume species (CC (13.6%), GM (12.0%), MT (18.3%), PV (14.7%), SL (17.4%), VA (14.6%), VR (14.7%), VU (15.9%), and VV (13.3%)), have more than 12% RLKs with this kinase domain modification. This RLK subset is interesting because it has been previously found that most PRR kinases or PRR-associated kinases have a change in a conserved arginine (R) located adjacent to the key catalytic aspartate (D) (the so-called RD motif) that facilitates phosphotransfer [39, 70].

Compared with RLKs, the majority of RLCKs reported in Table 3 only contain a Ser/Thr-specific cytoplasmic kinase domain, corresponding to previously reported results [71]. However, non-target domains were identified, contrary to the additional domains previously reported, which suggests that apart from the Pkinase, the RLCK could have similar intracellular domains as the ectodomains present in the RLKs, such as leucine rich repeat (LRR), lectin, epidermal growth factor (EGF), a domain of unknown function (DUF), U-BOX, and WD40 [71]. With the exception of the non/legume VV (4.7%), all other species [(AT (16.4%), CC (22%), GM (18.9%), MT (15.9%), PV (15.9%), SL (16.6%), VA (18.4%), VR (17.7%), and VU (14.82%)] had more than 15% of the RLKs classified as RLCKs. This is important because a number of RLCKs have emerged as central components linking PRR to downstream defenses. These PRRs are involved in transducing signals from extracellular ligands by phospho-relay [72]; several *Arabidopsis* RLCKs are associated with PRRs and play important roles in PTI [73].

The number of RLKs per species reported is proportionally similar to the 1 to 2% of total gene models per species reported in previous studies, where RLKs normally represented about 60% or more of protein kinases [37, 38]. The range of RLK proteins identified in this study was 450–1867 for legume proteins and 444–556 for non-legume proteins. The legumes GM (1867 proteins) and MT (1062 proteins) showed the highest number of RLKs. In contrast, the range for legume RLP proteins was 141–466 proteins and 160–170 for non-legume proteins. As with RLKs, the legumes GM (466 proteins) and MT (363 proteins) showed the highest number of RLPs.

Given that the RLK receptor configuration arises from a fusion between an RLP and an RLCK [74], it could be expected that RLPs have similar ectodomains, excluding the LRR and LysM domains that are experimentally reported for RLPs. The presence of other extracellular domains, which are mainly associated with RLKs, was explored to identify probable RLPs with the presence of L/C/G-lectin, TNFR, thaumatin, WAK, malectin, EGF, or stress-antifung domain. This approach was based on the similarities reported among two-plasma membrane receptors and suggests a consistent functional relationship and the possibility of novel domain configurations created by their fusion [35]. This approach discovered that for legumes (0.29 to 0.69%)/non-legumes (0.46 to 0.64%), less than 1% of the proteins present in the genomes belong to the RLP class.

Even though the TNFR domains belonging to both plasma membrane classes were not identified, a detailed evaluation showed that in the prediction process step (Pfam31, HMMER3.1, and PfamScan.pl), the domain match was considered insignificant because the bit score fell below the software threshold. However, RLK proteins have been predicted as RLKs with a TNFR extracellular domain and reported in the SMART database in an earlier study [75] for AT (2 proteins)*,* GM (4 proteins)*,* SL (2 proteins), and VV (3 proteins). Interestingly, with the exception of the VV proteins*,* the eight other proteins were identified as RLKs either with non-target domains or only the Pkinase domain. Other missed domains could include L-Lectin and TNFR for RLPs. This exploration of missing domains suggests that including tools such as SMART could add precision to the predictions in some instances.

Regarding the diverse domain combinations identified for RLK and RLP, RLK, in particular, vary greatly in their extracellular domain organization. A variety of extracellular domains are present in RLKs [16] such as LRR/Malectin; the S-locus/WAK present only in the legume VA and the non-legume VV; the B-lectin/PR5K present only in the legumes GM, MT, VA, and VU; the B-lectin/S-locus/WAK present only in the legumes CC, MT, PV, VA, and VU; and the B-Lectin/S-locus/Pan/WAK shared among the legumes GM, MT, PV, VA, and VU, and non-legumes VV and SL. The unique non-common ectodomain combination identified in RLP was LRR/Malectin, which was present in all species evaluated. This suggests the RLK domain combinations are more diverse compared with RLP combinations. Some RLK domain combinations were only reported for legumes, while RLP combinations were present among legumes and non-legumes.

The diversity of the Pfam domains to characterize various RLK and RLP as input criteria for classification is an advantage over using only target specific motifs [76]. Diversity of the Pfam domains was most evident in the RLK class for the Pkinases which possessed 10 domains/families. Among the 10 Pkinase domains/families, WaaY in MT; APH in PV, VA, VR and VU; and Pkinase_C in CC and GM were exclusively present in the legumes. For the 7 RLK-LRR, the LRR_2 was exclusively present in the legumes GM, VA, and VU. For other family domains, the EGF domain was only present in the legumes GM and MT. In contrast, for the ectodomains present in RLPs, the LRR_9 from the LRR clan was only present in CC; the L-lectin clan with the LPRY domain and the PAN clan with the PAN_4 domain were exclusive to all the legumes. Interestingly, those clans are collectively judged likely to be homologous and are valuable because they are built manually and integrate a diverse variety of information sources that allow the transfer of structural and functional information between families and improving the prediction of structure and function of unknown families [77]. The classification of non-target domains present for RLK and RLP among the species demonstrated that none of the most prevalent domains identified (present in 10-species) in both plasma membrane classes was common, suggesting a bias related to the kind of plasma membrane relation. This suggests that further analysis could be done to explore probable correlations among the domains evaluated.

## Conclusions

The identification of RLK and RLP based on the use of different machine-learning tools publicly available for the prediction of different biological features, allowed this study to propose a simple, logical, and effective set of conditions. The validation demonstrated that the approach is highly effective in identifying RLK/RLP proteins. The domains organization of RLK was more diverse compared with the domain organization of RLP domains. More L-lectin domain diversity exists in RLP (8 domains) compared with RLK (1 domain). Specifically, for the RLK, the non-RD represented 8 to 18%, and the RLCK represented about 15% of this class of plasma membrane proteins per species evaluated. Regarding the legume/non-legume comparison, *G. max* contains a larger set of RLK (1867 proteins) and RLP (466 proteins) compared with the legume/non-legume species. Across all species, the LRR ectodomain class was the most frequent domain per species. C-lectin is a rare domain commonly reported only once per genome, and only the GM species showed more than one such protein, which could be related to the recent whole genome duplication. For RLKs/RLPs among legumes/non-legumes, the LRR/Malectin domain combination is the most frequent among the dual combinations.

## Methods

### Independent evaluation of predictive performance

To evaluate the RLK and RLP prediction strategy, we test the ability to correctly classified or reject RLK, RLP, and non RLK/RLP proteins. The prediction performance used three evaluation sets with known outcomes supported by experimental evidence (Additional file 1: Table S1). For the performance evaluation measurement, sensitivity (range: 0 to 1), specificity (range: 0 to 1), and Matthews correlation coefficient “MCC” (range: − 1 to 1) were selected [50]. In the evaluation datasets, the identification of experimentally-validated proteins for each class became the true positive (RLP and RLK) and true negative data (cytoplasmic resistance genes) [50]. The cytoplasmic resistance genes could have similar ectodomains to RLK/RLP but have an exclusively NB-ARC domain [78]. The datasets obtained were independently processed using CD-HIT [79] to obtain a non-redundant version using a 90% identity to avoid similar or highly similar overlapping entries [50]. The predictive analysis of RLK/RLP was applied to the non-redundant sets; for the RLK evaluation, the RLP and “cytoplasmic resistance genes” sets were used as true negative proteins; for the RLP evaluation, the RLK and “cytoplasmic resistance genes” sets were used as true negative proteins.

### Genome dataset

To evaluate the proposed RLK/RLP identification strategy, three datasets were used (RLK, RLP and cytoplasmic resistance genes). All the datasets contain experimentally-validated proteins from 34 plant species (Additional file 1: Table S1) and were extracted from the UniProt Consortium [80]. The RLK set contained 66 proteins, the RLP set contained 28 proteins, and the set of cytoplasmic resistance genes (non-RLK/RLP), contained 96 proteins (Additional file 1: Table S1) [3, 43, 72, 73, 81]. To identify probable RLK and RLP, the analysis focused on seven legumes and three non-legumes (outgroup set), including *V. vinifera* because it represents the basal rosid lineage and has a close-to-ancestral karyotypes that facilitate comparisons across major eurosids [66, 67]. Also, non-legumes *Arabidopsis* and *S. lycopersicum* were included because they are model plants that could allow us to evaluate conservation and divergence. The protein information of the legumes/non-legumes is reported in Table 8.

**Table 8:** Summary of genomes

Species	Database	File name	N. of genes	N. of proteins	N. of chr
VR	NCBI	GCF_000741045.1_Vradiata_ver6	34,911	35,143	11
CC	NCBI	GCA_000340665.1_C.cajan_V1.0	23,374	48,331	11
VA	NCBI	annotation release 100	22,276	37,769	11
GM	Phytozome	gmax_275_wm82.a2.v1	55,589	88,647	20
MT	Phytozome	Mtruncatula_285_Mt4.0v1	48,338	62,319	8
PA	Phytozome	Pvulgaris_442_v2.1	27,012	36,995	11
VU	Phytozome	Vunguiculata_469_v1.1	28,881	42,287	11
AT	Phytozome	Athaliana_167_TAIR10	27,206	35,386	5
SL	Phytozome	Slycopersicum_390_ITAG2.4	33,838	34,725	12
VV	Phytozome	Vvinifera_145_Genoscope.12X	23,647	26,346	19

### Computational identification of RLK and RLP

The computational strategy for RLK and RLP discovery is described in Fig. 1. The identification of the presence/absence of signal peptide and transmembrane helices was predicted with SignalP 4.0 [62] and TMHMM 2 [63], respectively. The cut-offs used were Eukaryotes (euk): euk SignalP-noTM networks: 0.45 and euk SignalP-TM networks: 0.50 [62]. The selection criteria for TMHMM2 were based on the identification of one or more transmembrane helices, which must exceed the expected number of amino acids (ExpAA) threshold; if this value is larger than 18, it is very likely to be a transmembrane protein or have a signal peptide [63]. In both prediction processes, cut-off values are reported by default.

The PfamScan (pfamscan.pl) script [82] was used to annotate the protein sequences against the Pfam 31.0 library using HMMER 3.1b1 [64]. The selection criteria to assign a protein to each modular organization classes were defined by PfamScan, which states if overlapping matches within a clan are detected, it will then only report the most significant, which will be the lowest E-value match within the clan [83]. In some cases, proteins belonged to two domain classes, but the redundant information was extracted in the counting process. To establish a domain cutoff for Pfam-A searches, the parameter used by default was based on the diverse set of domains to reach these trusted cut-offs, which were defined by Pfam curators and their variable for each domain or family [64].

The PfamScan output was filtered using in-house scripts (https://github.com/drestmont/plant_rlk_rlp/) for the identification of RLK/RLP and their structural domains. The identification of the modular organization domains (Additional file 7: Table S7) is defined in the Pfam database [84] as profiles and clans (labelled: CL); the clans are profiles grouped together with a common evolutionary ancestor [82]. The in-house script includes 134 Pfam domains representing the extra domains and the Pkinase reported in Additional file 7: Table S7. They are considered “target domains” for this research and are reported in Additional file 6: Table S6 and Additional file 8: Table S8. The target clan or domain Pfam ID are reported in Table 9.

**Table 9:** Target domains for the classification of RLK/RLP

Functional family^a^	Clan or Domain	Number of domains reported in Pfam 31
LRR	CL0022	11
	LRRNT_2	1
Pkinase	CL0016	35
	Pkinase_C	1
L-Lectin	CL0004	43
C-Lectin	Lectin_C	1
G-Lectin	B_lectin	1
	S_locus_glycop	1
LysM	LysM	3
PR5K	Thaumatin	1
TNFR	TNFR	6
PAN	CL0168	6
WAK	WAK	1
	GUB_WAK	1
	WAK_assoc	1
Malectin	CL0468	2
EGF	CL0001	18
Stress-antifungal	Stress-antifungal	1
NB-ARC	NB-ARC	217

^a^Source: Pfam 31.0 [85]. The domains reported in Table 9 are not exclusively present on RLK and RLP. The NB-ARC belong to R genes, which belong to cytoplasmic proteins and were used to exclude false positive proteins

The identification approach follows this logic (logical operators: and, or, and not) (Fig. 1) for RLK: “presence/absence Signal peptide” and “transmembrane helix (at least one)” and “Pkinase domain/s” and “Extracellular domain/s: LRR or L-Lectin or C-Lectin or G- Lectin or LysM or PR5K or TNFR or WAK or Malectin or EGF or Stress-Antifung” not “NB-ARC” domains and, for RLP: “presence/absence Signal peptide” and “transmembrane helix (at least one)” and “Extracellular domain/s: LRR or L-Lectin or C-Lectin or G-Lectin or LysM or PR5K or TNFR or WAK or Malectin or EGF or Stress-Antifung” not “Pkinase domain/s” and not “NB-ARC domains”. Finally, a summary of the domain and family prevalence among species was obtained based on the RLK/RLP identified in the evaluation set and the species explored. The frequency analysis was based on the evaluation of “experimentally-validated protein datasets” (Additional file 1: Table S1), and also for the identified proteins, which belong to the species evaluated. After the RLK proteins per species were classified to identify potential non-RD proteins, the entire set of Pkinase sequence domains was broken into subsets using the start and end domain coordinates reported by PfamScan. The MEME command line tool version [86] was used to identify the RD and non-RD motif sites, and the MEME parameters used were as follows: -mod oops -maxw 10 -nmotifs 4 -maxsize 6,000,000. After the motif sites were reported, they were classified as RD ([H][R][D]) and non-RD ([H][^R][D]) motif (regex notation). The kinome was identified by annotating the whole set of proteins per species using pfamscan.pl. The proteins with the presence of Pkinase domains were filtered (Table 2 – footnote and Additional file 3: Table S3).

## Supplementary information

**Additional file 1: Table S1.** Experimentally-validated RLK, RLP, and R gene proteins used to evaluate the prediction.

**Additional file 2: Table S2.** Performance prediction evaluation of RLK and RLP reported on Table S1

**Additional file 3: Table S3.** RLK-nonRD IDs identified among the species evaluated

**Additional file 4: Table S4.** Protein ids of the 10 species evaluated that are classified as RLK (psg: presence of signal peptide and nsg:absence of signal peptide).

**Additional file 5: Table S5.** Protein ids of the 10 species evaluated that are classified as RLP (psg: presence of signal peptide and nsg:absence of signal peptide).

**Additional file 6: Table S6.** Specific domains that belong to the clans and families to classified RLP and RLK proteins.

**Additional file 7: Table S7.** Modular organization of RLK and RLP in plants.

**Additional file 8: Table S8.** Summary of the clans and domains reported on Pfam 31.0.

**Additional file 9: Table S9.** Remaining domains identified in the RLK among the species evaluated.

**Additional file 10: Table S10.** Remaining domains identified in the RLP among the species evaluated.

## Data Availability

All data analyzed during this study are included in Phytozome, the NCBI and Pfam database. The datasets generated and/or analyzed during the current study are available in the github repository, https://github.com/drestmont/plant_rlk_rlp/

## Abbreviations

RLK: Receptor-Like Kinase
RLP: Receptor-Like Protein
RLCK: Receptor-Like Cytoplasmic Protein Kinase
RLK-nonRD: Receptor-Like Kinase with absence of an arginine (R) located before a catalytic aspartate (D)
NB-ARC: Nucleotide-Binding domain shared by plant resistance gene products
R genes: Resistance genes
PRR: Pattern Recognition Receptors
MAMP: Microbe-Associated Molecular Patterns
PAMP: Pathogen- Associated Molecular Patterns
DAMP: Damage-Associated Molecular Patterns
MCC: Matthews Correlation Coefficient
NB-LRR/NBS-LRR: Nucleotide Binding Site-Leucine-Rich Repeat proteins
PGIP: Polygalacturonase-Inhibiting Protein
LRR: Leucine-Rich Repeat
C-Lectin domain: Carbohydrate-binding protein domain
G-Lectin domain: S-receptor-like or S-locus
L-Lectin domain: L-like lectin domain
LysM: Lysin Motif
NFR: Nod factor receptor
LYK3: Putative *Medicago* ortholog of NFR1
FER: Feronia or protein Sirene
PR: Pathogenesis-Related
TLP: Thaumatin-Like proteins
WAK: Cell-wall-associated kinases
EGF: Epidermal Growth Factor-like repeats
TNF/TNFR: Tumor necrosis factor/tumor necrosis factor receptor superfamily
Stress-Antifung domain: Formerly known as DUF26 (Domain of Unknown Function)
Kinase domain RD: Presence of an arginine (R) located before a catalytic aspartate (D) residue
Kinase domain non-RD: Absence of an arginine (R) located before a catalytic aspartate (D) residue
GM: *Glycine max*
PV: *Phaseolus vulgaris*
MT: *Medicago truncatula*
VR: *Vigna radiata*
VU: *Vigna unguiculata*
VA: *Vigna angularis* var. *Angularis*
CC: *Cajanus cajan*
AT: *Arabidopsis thaliana*
SL: *Solanum lycopersicum*
VV: *Vitis vinifera*
